# CuO-Ga_2_O_3_ Thin Films as a Gas-Sensitive Material for Acetone Detection

**DOI:** 10.3390/s20113142

**Published:** 2020-06-02

**Authors:** Katarzyna Dyndal, Arkadiusz Zarzycki, Wojciech Andrysiewicz, Dominik Grochala, Konstanty Marszalek, Artur Rydosz

**Affiliations:** 1 Department of Electronics, AGH University of Science and Technology, Al. Mickiewicza 30, 30-054 Kraków, Poland; kkoper@agh.edu.pl (K.D.); marszale@agh.edu.pl (K.M.); 2 Institute of Nuclear Physics Polish Academy of Sciences, PL-31342 Kraków, Poland; arkadiusz.zarzycki@ifj.edu.pl; 3 CBRTP SA, Waryńskiego 3A, 00-645 Warszawa, Poland; andrysiewicz@agh.edu.pl; 4 Department of Biocybernetics and Biomedical Engineering, AGH University of Science and Technology, Al. Mickiewicza 30, Al. Mickiewicza 30, 30-054 Kraków, Poland; grochala@agh.edu.pl

**Keywords:** gas sensors, acetone detector, thin-films, copper oxides, gallium oxides

## Abstract

The p-n heterostructures of CuO-Ga_2_O_3_ obtained by magnetron sputtering technology in a fully reactive mode (deposition in pure oxygen) were tested under exposure to low acetone concentrations. After deposition, the films were annealed at previously confirmed conditions (400 °C/4 h/synthetic air) and further investigated by utilization of X-ray diffraction (XRD), X-ray reflectivity (XRR), energy-dispersive X-ray spectroscopy (EDS). The gas-sensing behavior was tested in the air/acetone atmosphere in the range of 0.1–1.25 ppm, as well as at various relative humidity (RH) levels (10–85%). The highest responses were obtained for samples based on the CuO-Ga_2_O_3_ (4% at. Ga).

## 1. Introduction

Gas detectors have constantly been developed over the last few decades, and the number of applications is still growing, from industrial to everyday life applications [1,2,3,4,5,6]. Generally, the gas sensors consist of few elements, such as active material, referred to as the gas-sensitive layer, and transducers, including electrodes, package, and front-end electronics. The gas-sensitive layers are based on different materials, and metal oxides are one of the most commonly used. Among them, copper oxide has become increasingly attractive. The copper oxide is a typical p-type semiconductor, it has been used as a base material for several applications, including solar energy cells [7], optoelectronics [8], catalysis [9,10], biosensors [11], supercapacitors [12], lithium ion batteries [13], electrochemical sensors [14], and gas sensors [15]. An actual review of CuO-based gas sensors was presented by the authors of [16], where various different deposition methods, target gases, and operating conditions were discussed. The CuO-based gas-sensing materials utilize a pure copper oxide, as well as copper oxide doped with various metals, e.g., Ag, Au, Cr, Pt, Sb, Si [17], Al [18], Fe, Li [19,20], Na [20], grapheme [21], Pd [22], Zn [23], and other oxides. Recently, the following compositions of copper oxide and other metal oxides (MOX) were reported, such as CuO/TiO_2_ [24], CuO/CuS [25], ZnO/CuO [26,27,28], CuO/In_2_O_3_ [29], CuO/NiO [30,31], WO_3_/CuO [32], rGO/CuO [33,34], PtO_2_/CuO [35], Fe_2_O_3_/CuO [36], CuO/SnO_2_ [37,38,39], and CuO/CeO_2_ [9,40].

The CuO-based gas sensors are used for detection of reducing gases and oxidizing gases, such as CO [41,42,43,44,45], H_2_ [23,45,46,47], NH_3_ [33,43], C_6_H_6_ and C_7_H_8_ [45,48], H_2_S [26,37,38,43], CH_3_OH and C_3_H_6_O [40], C_2_H_5_OH [43,49], and NO_2_ [41,43,45,50,51,52], SO_2_ [43,45], O_2_ [45], and CO_2_ [43], respectively. The responses are usually defined as the resistance ratio R_a_/R_g_ or R_g_/R_a_, where R_a_ and R_g_ are electrical resistances in air and target gas, respectively. Apart from the common compositions mentioned above, a CuO-Ga_2_O_3_ has not been reported to date. Therefore, in this paper, a CuO-Ga_2_O_3_-based gas sensor for enhanced acetone detection was presented. 

Ga_2_O_3_-based gas sensors have been reported since 1991 [53], and since then, gallium oxides have been studied as potential films in gas-sensing applications. There are several polymorphs of Ga_2_O_3_ [54], such as the rhombohedral (α-Ga_2_O_3_), monoclinic (β-Ga_2_O_3_), defective spinel (γ-Ga_2_O_3_), cubic (δ-Ga_2_O_3_), and orthorhombic (ε-Ga_2_O_3_) structure, among which the most chemical and thermal stable under ambient conditions is β-polymorph [55]. Moreover, the various forms of low-dimensional crystalline Ga_2_O_3_, including nanowires (NWs) [56,57], nanobelts (NBs) [58], and nanosheets (NSHs) [59], are the subject of research. Ga_2_O_3_ is an n-type semiconductor with an ultrawide-bandgap in the range of 4.2–5.3 eV [54,60,61]. Ga_2_O_3_ is an interesting material due to its high conductivity [62,63,64], optical transparency in ultraviolet and visible regions [65], and excellent photoluminescence property, which is a result of its defect-rich structure [66]. Ga_2_O_3_ sensors show fast response and recovery times, good reproducibility, low cross sensitivity to humidity, and short pre-ageing times [67]. Moreover, the Ga_2_O_3_ has found a wide range of applications in various disciplines. Thin layers of gallium oxides have been used in solar cells as an ultrathin tunneling layer in the dye-sensitized solar cell, as well as in passivation layers on silicon solar cells, photodetectors [68,69], and electronic devices [70]. Also, a lot of reported works have focused on gas-sensing behaviors based on this metal oxide, as well as its doped-Ga_2_O_3_ composite (i.e., Sn [71], Ce, Sb, W, Zn [72,73], Sn, Cu, N [74], Au [75]) and metal oxide/Ga_2_O_3_ composite (i.e., TiO_2_ [76], SiO_2_ [77], SnO_2_ [78], I_2_O_3_ WO_3_ [79,80], ZnO [81], MgO [82]). Gas sensors based on Ga_2_O_3_ are used for gas detection, e.g., C_2_H_5_OH and C_3_H_5_OH [79,83], C_2_H_4_ [72], H_2_ [61,63,79], O_2_ [72,84], NO_2_ [76], CO [76,79,85], and NH_3_ [79,86,87].

Several techniques were used to deposit or grow Ga_2_O_3_ thin films, e.g., vapor-vapor-liquid-solid [62], chemical vapour deposition [88], spray pyrolysis [89], molecular beam epitaxy [90], electronic beam evaporation [91], pulsed laser deposition [92], magnetron sputtering [68], sol-gel [93] and vacuum thermal evaporation [66], plasma-enhanced atomic layer deposition [65], and atomic layer [94].

As previously mentioned, CuO-Ga_2_O_3_ is a p-type/n-type heterostructure, where the base material (CuO) has bandgap in the range 1.2–1.9 eV depending on the crystalline phase and stoichiometry [95,96], and the upper layer (Ga_2_O_3_) has been reported in the range 4.2–5.3 eV of the bandgap. The application of p-type CuO and n-type ZnO heterostructure is well known with all of the advantages in gas sensor technology. The system designed for this study looked very similar and had similar advantages and similar improvement in sensing performance, especially for higher Ga_2_O_3_ content. Electronic effects, such as the charge carrier separation or increased interfacial potential barrier energy of such system, are affected by the electronic properties of the system, leading to better sensitivity of the sensor on the sub-ppm level.

The Ga_2_O_3_-based gas sensors are shown in Table 1. For example, the authors of [83] investigated the impact of air humidity on the detection of gases such as H_2_, C_2_H_4_, and C_3_H_5_OH. The tests were carried out for 200 ppm and 500 ppm concentrations of H_2_, C_3_H_5_OH, and C_2_H_4_, respectively, in dry and wet air (15% RH) at temperatures in the range from 480 °C to 640 °C. C_3_H_5_OH studies have indicated that the sensitivity is higher in dry air compared to wet air and decreases as the temperature increases. Similar behaviors were observed for C_2_H_4_. For acetone, the sensor response was 17 and 11 (resistance ratio) at 550 °C in dry and wet air, respectively. In contrast, for the same temperature, the sensor response to H_2_ was 24 and 15 for dry and wet air. The obtained results for the H_2_ indicate that the presence of moisture at temperatures below 600°C inhibits the sensing reaction. In [79], Paul et al. presented a comparison of Ga_2_O_3_-core/WO_3_-shell nanostructures sensor responses with Ga_2_O_3_ to such gases as C_2_H_5_OH, C_3_H_5_OH, NH_3_, CO, and H_2_. For each gas, the sensitivity of the sensor was higher for Ga_2_O_3_-core/WO_3_ compared to pure Ga_2_O_3_. The author described this effect as a combination of the potential barrier–controlled carrier transport mechanism and the surface-depletion mechanism.

## 2. Materials and Methods

### 2.1. Gas-Sensitive Layer Deposition

The gas-sensitive layers were based on copper oxide, which was previously confirmed as good gas-sensitive material [16,17,100], as well as on the copper oxide–gallium oxide composition with various Ga_2_O_3_ contents. The films were deposited by the magnetron sputtering system with a glancing angle deposition technique and mosaic sputtering, where pure Ga (99.99999% purity) metal were placed on the pure Cu (99.9999%) magnetron target. The deposition conditions were previously fixed and are briefly presented in the Table 2. 

### 2.2. Gas-Sensing Measurements

**Figure 1:**
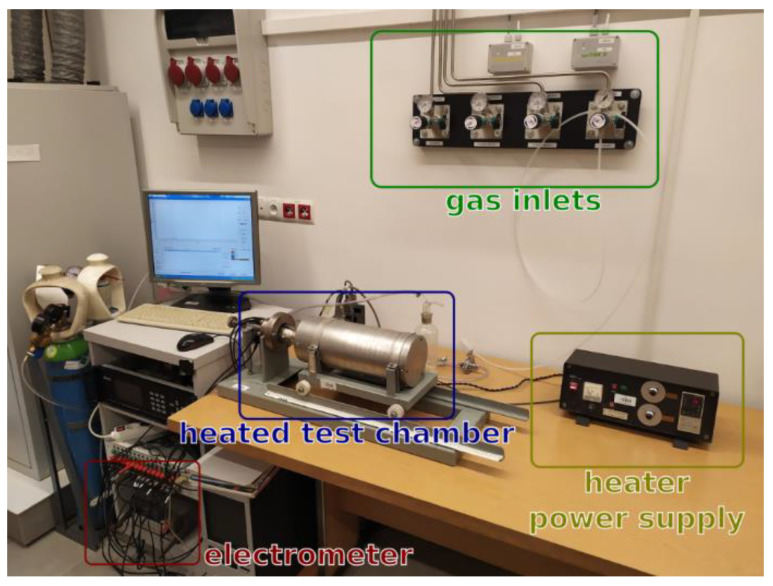
The measurement setup for gas-sensing characteristics. Reprinted from [101] CC BY 4.0.

The gas-sensing measurement system consisted of a few elements: The quartz-tube oven with an internal heater and temperature control unit, gas-dosing lines equipped with mass flow controllers 1179B (MKS Instruments, Andover, MA, USA), and the resistance measurement unit, which consisted of an electrometer (34401A HP, Keysight, MA, USA) and target gas canisters with 5 ppm of acetone (Air Products, Hersham, UK). The gas-sensing measurement system was previously described in detail [101] The gas-sensing measurements were performed at various temperatures and 50% relative humidity (RH) level. Figure 1 shows the measurement system. The gas sensor response (S) was defined as the resistance ratio S = R_gas_/R_air_, where R_gas_ and R_air_ are electrical resistances in gas and air, respectively. 

### 2.3. EDS, XRD and XRR

The chemical composition of the samples was studied with scanning electron microscopy (Vega Tescan 3, Vienna, Austria) equipped with an X-ray spectroscopy (EDS) spectrometer (Bruker XFlash 610M, Vienna, Austria). For each sample, the EDS spectroscopy measurements were done for several arbitrary points, which were chosen on the surface of the sample. Additionally, maps with a scan size of around 10 × 10 μm were performed to study elements distribution. The acquisition time for single-point measurement and map were 5 min and 1 h, respectively. 

The X-ray diffraction (XRD) and X-ray reflectivity (XRR) measurements were performed with an X’PertPro PANalitycal diffractometer equipped with an X-ray source with a Cu anode operating at 40 kV and 30 mA. The XRR data were collected for the ω angle between 0.2–2.0 degrees for 12 h for a sample to ensure sufficient statistics. The XRD θ/2θ patterns were gathered for a 2θ angle between 30 degrees and 90 degrees for 12 h for each sample. Additionally, in order to reduce signal from the Si wafer, a scattering vector was tilted by an angle of 3 degrees with respect to the diffraction plane. Details about measurement conditions have been described by the authors of [102,103]. The analysis of the obtained diffraction patterns was performed with FullProf software [104].

## 3. Results and Discussion

### 3.1. Characterization 

#### 3.1.1. SEM

The EDS chemical composition analysis showed the presence of Si, O, Cu, and Ga elements in all samples, together with signals from C and N arising from sample contamination from the air. From the point of view of this study, the relative composition of copper and gallium was the most important factor. Hence, in this study, EDS analysis was restricted to those elements only. Table 3 shows the relative atomic compositions for Cu and Ga elements.

**Figure 2:**
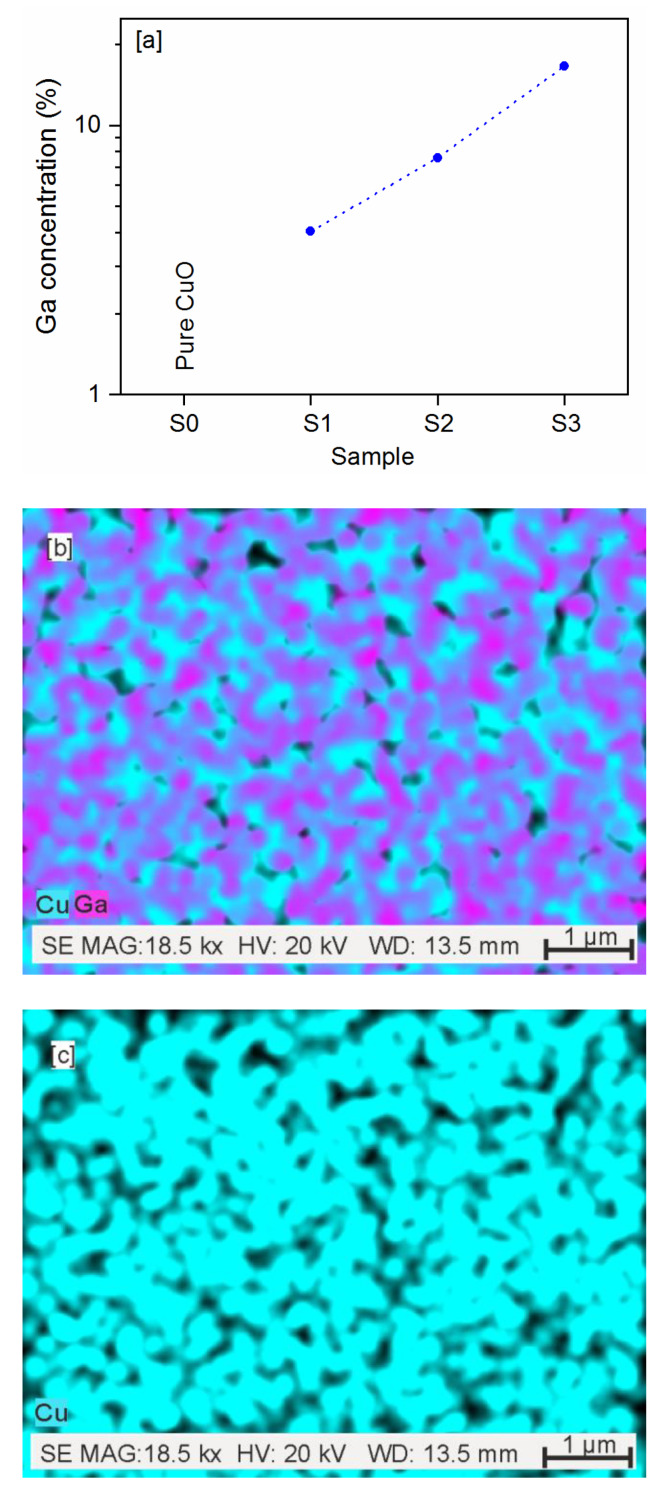

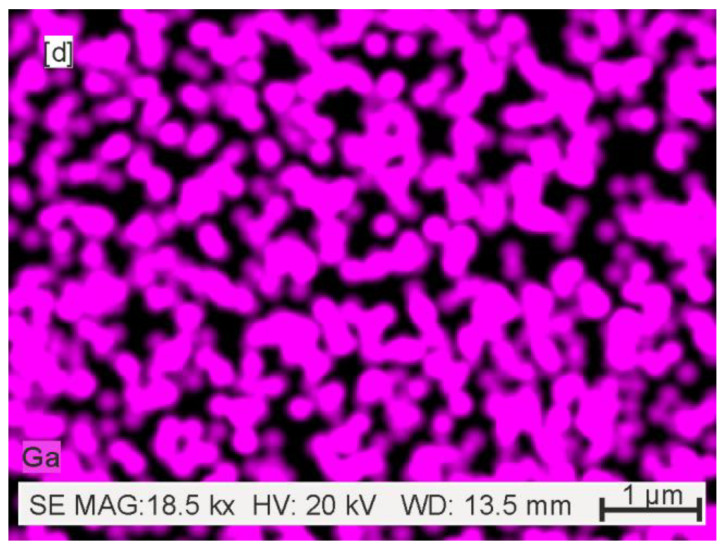
Gallium concentration (**a**), distribution map of sample S3 for Cu and Ga elements (**b**), and distribution maps of only Cu (**c**) and only Ga (**d**) elements for the same sample.

Figure 2a shows an exponential increase of gallium concentration between the samples, where the concentration changed between ~4 at.% and ~16 at.%. The Ga concentration in the sample was doubled as compared to the previous sample. A typical distribution of Cu and Ga elements is presented in Figure 2b based on the sample S3. In Figure 2b, blue indicates Cu, and magenta indicates Ga. The map confirms the homogeneous distribution of both elements and a higher concentration of copper as compared to gallium. Because there were no visible differences in elements distribution between samples containing different amount of gallium, only maps for the sample with the highest Ga concentration are presented in the paper, and all distributions are included in the Supplementary Materials Section.

#### 3.1.2. XRD/XRR

**Figure 3:**
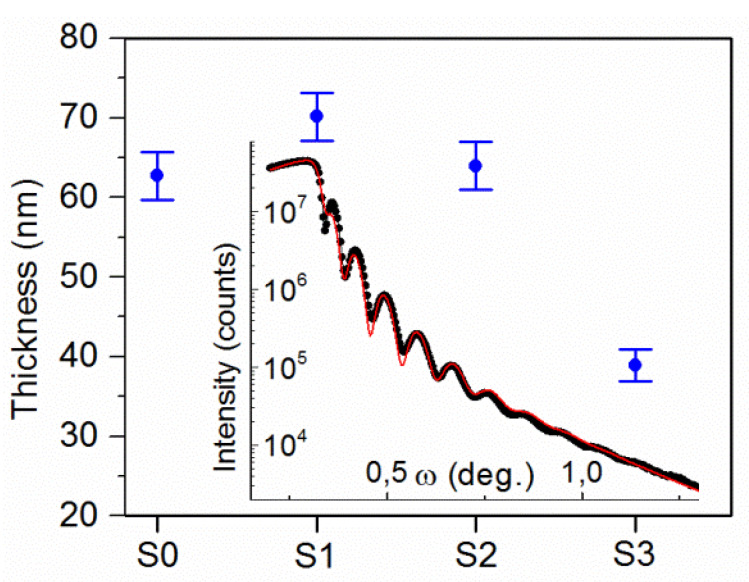
Films thicknesses for samples with different Ga concentration. In the insert, the XRR spectrum obtained for sample S4 with about 16 at.% of gallium is shown.

The X-ray reflectometry analysis showed that the samples had a thickness in the range of 60–70 nm, except the sample with the largest concentration of Ga, where the layer thickness was 40 nm. The various thicknesses were the consequence of the deposition time, which was kept constant (20 min) to kept the same deposition parameters for various gallium metal dopants placed on the copper magnetron target. The obtained results are presented in Figure 3 together with the spectra gathered for CuO:Ga_2_O_3_ (sample S4, shown as an insert). The density in all studied cases was found to be around 5.6(1) g/cm^3^ regardless of the gallium concentration. This value was significantly lower than that found for bulk copper(II) oxide, which was 6.31 g/cm^3^. Similarly, the lack of dependence of roughness with film composition was observed. The mean value was found to be relatively large, at around 3 nm.

**Figure 4:**
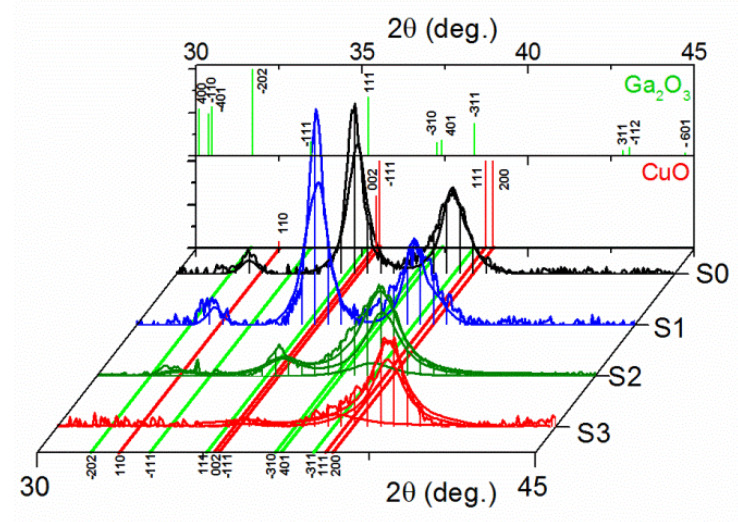
Diffraction patterns of CuO:Ga samples with fits and with marked positions of Braggs maxima for bulk CuO and β-Ga_2_O_3_ phases (back panel).

The diffraction patterns for all samples are presented in Figure 4. The continuous lines are the measurements results (thin irregular lines) together with fits (thicker and smooth lines). The longitudinal red and green lines indicate the position of Braggs maxima for the CuO and β-Ga_2_O_3_ phases, while the back panel presents the powder X-ray diffraction patterns of those oxides. Both phases had a monoclinic crystal system with the C2/c and C2/m space group, respectively (ICSD card No. 00-045-0937 and ICSD card No. 00-041-1103). During the analysis, for the two samples with the smallest Ga amounts, only one phase of copper oxide was fitted, while for samples with about 8 at.% and 16 at.% of gallium, a small amount of β-Ga_2_O_3_ was also found, meaning a sufficient signal from crystalline gallium(III) oxide was detected with XRD measurements. For those diffractograms, three fitting lines were included on the graph, indicating patterns for the CuO and β-Ga_2_O_3_ phases and a total phase. Additionally, for samples dopped with the largest amount of gallium, a shift in preferential grain orientation was clearly evident. The wide and intensive maxima observed for an angle around 35 deg., in case of sample S1 (CuO:Ga_2_O_3_ with 4% at. Ga), became weak and small in samples strongly dopped with gallium. Furthermore, a change in the pattern leading to a shift of the peak was found around 38 deg. due to the larger values and its separation from at least two individual Bragg maxima.

**Figure 5:**
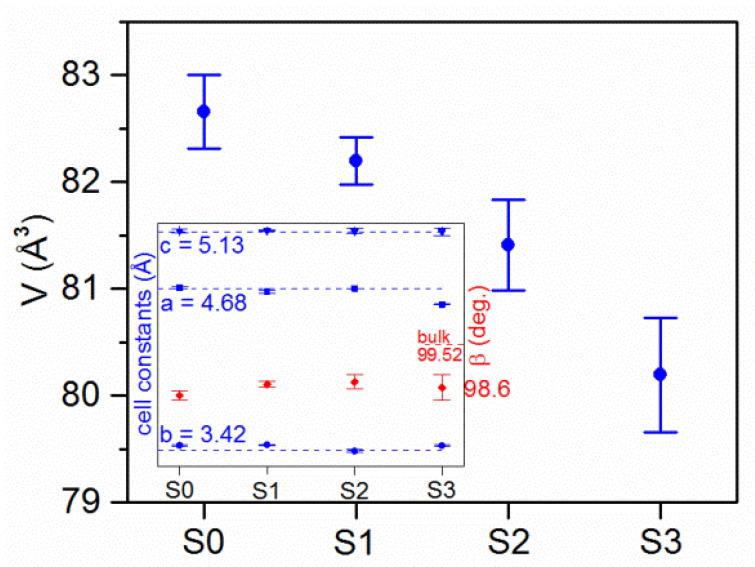
Changes in cell volume of CuO phase with an increase of gallium concentration. Insert presents the cell parameters of copper oxide.

Because of small amount of gallium oxide in the pattern, the crystallographic parameters used for this phase during the fitting procedure were from the crystallographic database and were kept constant during analysis. On the other hand, the parameters for copper oxide were varied and slow, and a systematic change with the increase of gallium concentration was observed. Figure 5 shows a change of cell volume with Ga concentration where a decrease from ~82 A3 to ~80 A3 is clearly seen. The insert of the graph shows dependence of cell parameters of a, b, c and β constants with gallium concentration. The dashed lines are values found in the database for the bulk sample. The most significant change was with the β angle, where a reduction of around 1 deg. was found for all studied samples, while the difference between the specific Ga concentration was uncertain. As mentioned previously, the reduction of cell volume is a consequence of the changes of the Ga concentration, which can be seen especially well for the sample S4 with the 16 at.% of Ga. Furthermore, with a shift of the a parameter, a strong (100) crystallographic texture was found in CuO:Ga_2_O_3_ films with 8 at.% (sample S2) and 16 at.% Ga (sample S3). Also, for those samples, an increase of peaks widths was found. 

An estimation of the size of crystallites was performed by applying a Scherrer formula. The Scherrer formula, linking coherence length (L_coh_) together with the width of Braggs maxima (ω) found at 2θ angle for used wavelength λ, allowed us to estimate the size of crystallites: L_coh_ = kλ/ωcos θ, where k is a Scherrer constant typically equal to 0.95 for thin films [102]. The samples S0 and S1 without and with small gallium concentration (i.e., pure CuO and CuO:Ga_2_O_3_ with 4% at. Ga) had a mean grain size of 12–14 nm, while the S3 and S4 samples with 8 at.% and 16 at.%, respectively, showed a decrease of mean grain size to ~7 nm. Furthermore, the mean size of gallium(III) oxide crystallites for those two samples was found to be even lower, at around 5 nm. The XRD analysis allowed us to estimate the amount of crystalline β-Ga_2_O_3_ phase in the spectra to be of several percents, i.e., 3(1)% and 5(1)% for S2 and S3 samples with Ga concentration of 8 at.% and 16 at.%, respectively.

#### 3.1.3. Gas-Sensing Characteristics

**Figure 6:**
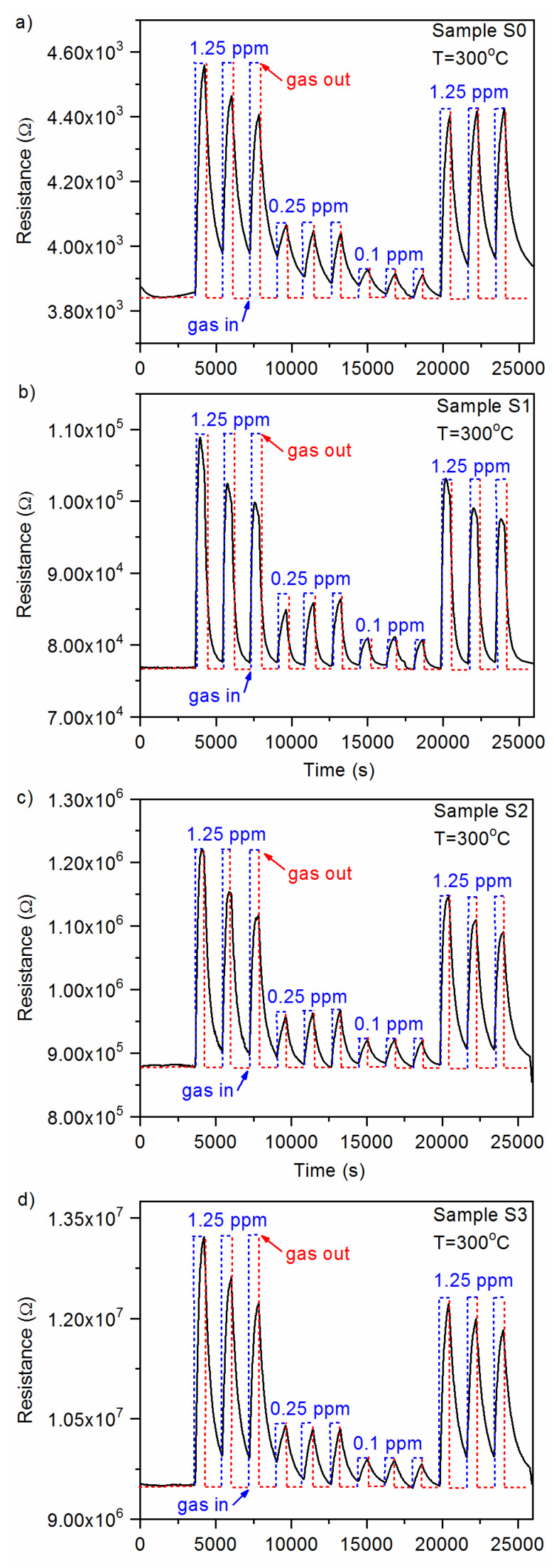
Sensor response as a function of acetone concentration at (**a**) 300 °C and 50% RH for CuO (sample S0), (**b**) CuO:Ga films deposited with 4% at. (sample S1); (**c**) 8% at. (sample S2); (**d**) 16% at. (sample S3) of gallium concentration.

**Figure 7:**
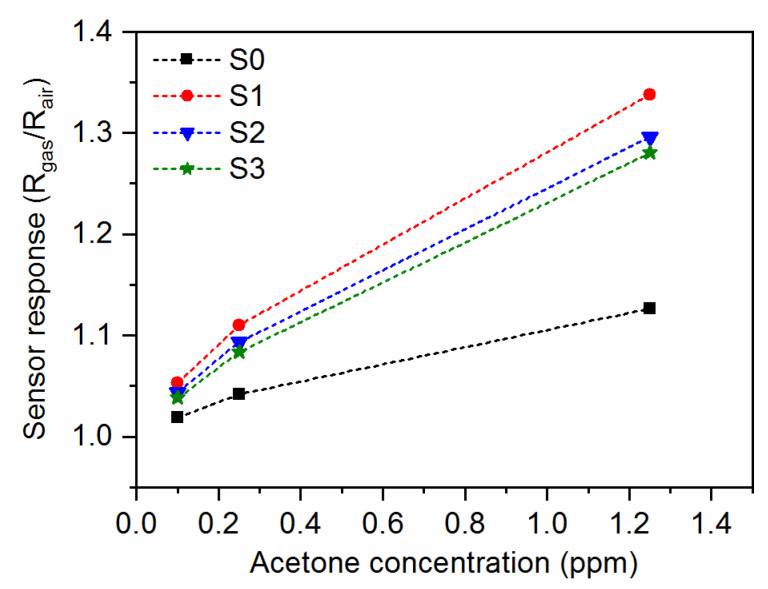
Sensor response as a function of acetone concentration at 300 °C and 50% RH for pure CuO (sample S0) and CuO:Ga films deposited with 4% at. (sample S1), 8% at. (sample S2), and 16% at. (sample S3) of gallium concentration.

**Figure 8:**
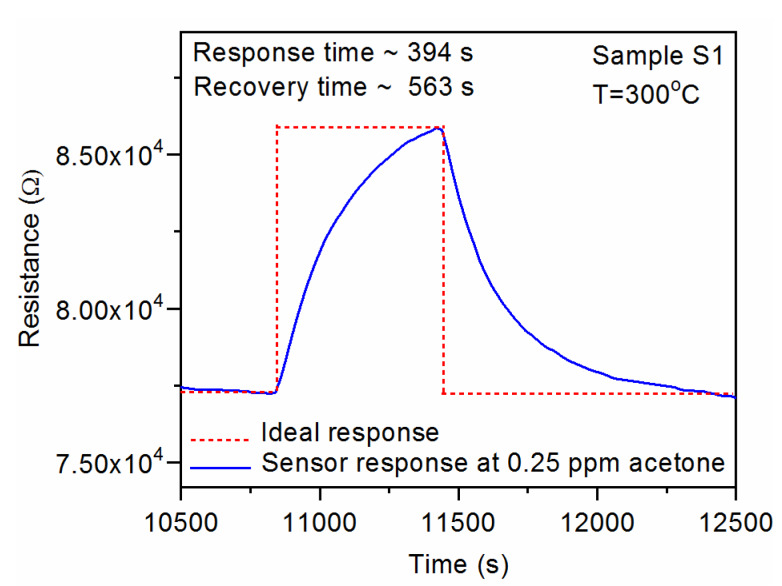
The response and recovery times at 300 °C for CuO:Ga_2_O_3_ films (sample S1) with ~4% at. Ga at the exposition to 0.25 ppm acetone.

**Figure 9:**
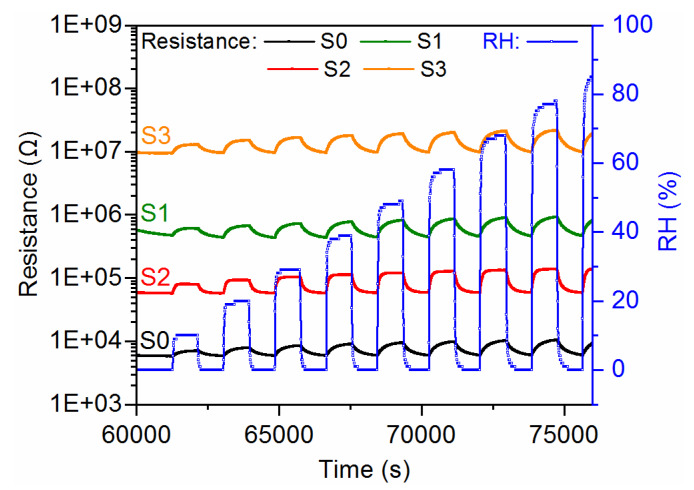
The resistance changes of the fabricated sensors S0–S4 measured at 300 °C at various relative humidity levels.

The gas-sensing characteristics of the CuO-Ga_2_O_3_-based gas sensors under exposure to various acetone concentrations in time function, with gas-in/gas-out phases, are presented in Figure 6a–d. As can be observed, the p-n heterostructure made of p-type CuO and n-type Ga_2_O_3_ reacted in increasing the resistance when acetone was introduced to the measurement cell and decreased the resistance in synthetic air. Therefore, the sensor response was defined as the resistance ratio R_gas_/R_air_, where R_gas_ and R_air_ are electrical resistance under exposure to acetone and synthetic air, respectively. The sensor response as a function of acetone concentration at 300 °C and 50% RH is presented in Figure 7. As can be noticed, the doping effect of Ga_2_O_3_ to pure CuO slightly increased the sensor response in the 0.1–1.25 ppm of acetone. However, the highest responses were obtained for samples with lower gallium oxide content, i.e., around ~4% at. Therefore, further investigations will be focused on the experiments with 2–6% at. doping. Moreover, sample S1 exhibited 40% faster response time in comparison with pure CuO samples (Figure 8). All response/recovery times are shown in Table 4. The resistance changes measured at various relative humidity concentrations are presented in Figure 9. The fact that the gas-sensing performance of thin film-based sensors is correlated with humidity is widely known. However, as can be noticed, the fabricated sensors remained stable in the full range of various relative humidity due to the p-n structure. The relative resistance changes in the 10–90% relative humidity range were around ~30% for all samples. However, in the range, 50–90% of changes were below 5% which makes the developed sensors very attractive for utilization in the highly humidified samples, such as exhaled human breath analysis.

#### 3.1.4. Response and Recovery Times

The response and recovery times are presented in the Table 4 as well as an example of the calculation is given in Figure 8. Briefly, in the gas-sensing applications, the response and recovery time (s) is defined as the time to reach a 90% variation of the sensor signal. In this study, resistance under exposure to target gas and air, respectively. It has to be underlined, that the obtained times are quite high and further works should be focused on the reduction of response and recovery time (s), for example by using noble metal dopings. 

## 4. Conclusions

The GLAD magnetron sputtering was used for CuO-Ga_2_O_3_ film deposition from the Cu:Ga mosaic target for gas sensor purposes. The sputtering conditions were presented for the thin CuO-Ga_2_O_3_ deposition with an average deposition rate of about 2.5 nm/min. The sample structure was investigated by XRD, XRR, and EDS measurements. The content of gallium was determined to be 4 at.% to 16 at.%, and film density was found to be 5.6 g/cm^3^ regardless of the gallium concentration. Diffraction patterns indicated Braggs peaks for the monoclinic phase of CuO for samples with a low Ga content, and CuO and β-Ga_2_O_3_ monoclinic phases for samples with a higher Ga content. Also, the grain size changed from around 7 nm for a low content of gallium to 12–14 nm for samples with a higher content of gallium. For the measurements of the sensing properties of the CuO-Ga_2_O3 system, all investigated samples were able to detect acetone on levels of 1.25 ppm, 0.25 ppm, and 0.1 ppm. The signal values are presented in Figure 8. The response and recovery times were high, however, it has to be underlined that the results were related to the measurement setup, in which a high volume quartz-glass tube was used. The advantage of this type of sensor is its low humidity influence on the signal level. In conclusion, according to the CuO-Ga_2_O_3_ system review and our results, the design was properly designed to detect very low level acetone concentration sensors on ppm and even below ppm levels.

## Figures and Tables

**Table 1:** The summary of the recently presented research results on gas-sensing applications.

Gas-Sensing Layer Material	Deposition Method	Target Gas	Concentration (ppm)	Response	Operating Temp. (°C)	Ref.
Ga_2_O_3_	screenprinting	C_3_H_6_O	200	17 ^A^/11 ^A^	550	[83]
Ga_2_O_3_-core/WO_3_	thermal evaporation	C_3_H_6_O	200	210 ^B^	200	[79]
Ga_2_O_3_	screenprinting	C_2_H_4_	500	15 ^A^	550	[83]
Ga_2_O_3_	thermal evaporation	C_2_H_5_OH	200	210 ^B^	200	[79]
Ga_2_O_3_-core/WO_3_	thermal evaporation	C_2_H_5_OH	200	510 ^B^	200	[79]
Ga_2_O_3_	sputtering techniques	C_2_H_5_OH	37	175 ^A^	800	[67]
Ga_2_O_3_	screenprinting	H_2_	500	24/15 ^A^	550	[83]
Ga_2_O_3_	vapor–liquid solid	H_2_	100	3.4 ^C^	300	[63]
Ga_2_O_3_	sputtering techniques	CH_4_	5000	7 ^A^	800	[97]
Ga_2_O_3_-core/WO_3_	thermal evaporation	H_2_	1000	310 ^B^	200	[79]
Ga_2_O_3_	thermal evaporation	H_2_	1000	110 ^B^	200	[79]
Ga_2_O_3_	thermal evaporation	NH_3_	100	160 ^B^	200	[79]
Ga_2_O_3_-core/WO_3_	thermal evaporation	NH_3_	100	200 ^B^	200	[79]
β-Ga_2_O_3_	drop casting method	NH_3_	100	50 ^D^	RT	[86]
β-Ga_2_O_3_	spray pyrolysis	NH_3_	20	2500 ^F^	30	[87]
Ga_2_O_3_	thermal evaporation	CO	100	120 ^B^	200	[79]
Ga_2_O_3_-core/WO_3_	thermal evaporation	CO	100	210 ^B^	200	[79]
Ga_2_O_3_	sputtering techniques	CO	5000	6 ^C^	650	[85]
Ga_2_O_3_/Al_2_O_3_	two-step hydrothermal and calcining method	NO_x_	70	55 ^B^	RT	[92]
Ga_2_O_3_/Al_2_O_3_	two-step hydrothermal and calcining method	NO_x_	70	7 ^B^	RT	[98]
Ti-O_2_-Ga_2_O_3_	sol-gel method	NO_2_	10	3.5 ^E^	200	[86]
Ga_2_O_3_	chemical thermal evaporation method	O_2_	5	10 ^G^	300	[99]
Ga_2_O_3_	chemical thermal evaporation method	CO	500	5 ^G^	100	[99]
TiO_2_-Ga_2_O_3_	sol-gel method	CO	400	7 ^E^	200	[76]

^A^ R_0_/(R_0_ + R_a_), ^B^ (R_0_/R_a_)·100%, ^C^ R_a_/R_0_, ^D^ (R_a_ − R_0_)/R_0_·100%, ^E^ R_0_/R_a_, ^F^ (R_0_ − R_a_)/R_a_·100%, ^G^ (R_a_ − R_0_)/R_0_, where R_0_ and R_a_ are the resistance in carrier gas and R_a_ the resistance in the target gas, respectively.

**Table 2:** The summary of the deposition conditions.

Parameter	Value
Base pressure	1 × 10^−^^6^ mbar
Deposition pressure	2 × 10^−2^ mbar
Target to substrate distance	60 mm
Deposition temperature	100 °C
Deposition mode	fully reactive, deposition at pure oxygen
Presputtering time	10 min
Presputtering power	100 W
Sputtering time	20 min
Sputtering power	50 W
Oxygen flow	20 sccm

**Table 3:** Atomic concentration of copper and gallium from X-ray spectroscopy (EDS).

CuO-Ga_2_O_3_	Cu at.%	dCu at.%	Ga at.%	dGa at.%
**S1**	95.93	3.05	4.03	11.24
**S2**	92.42	3.08	7.58	7.60
**S3**	84.45	3.315	16.55	5.10

**Table 4:** Response and recovery times obtained for CuO and CuO:Ga films with different gallium concentrations at 1.25 ppm, 0.25 ppm, and 0.1 ppm acetone exposure.

Acetone → Concentration		1.25 ppm		0.25 ppm		0.1 ppm	
Samples ↓		t_response_ [s]	t_recovery_ [s]	t_response_ [s]	t_recovery_ [s]	t_response_ [s]	t_recovery_ [s]
**S0**	CuO	319	901	431	862	451	825
**S1**	CuO:Ga_2_O_3_ (~4% at. Ga)	187	525	394	563	394	601
**S2**	CuO:Ga_2_O_3_ (~8% at. Ga)	264	658	413	752	489	884
**S3**	CuO: Ga_2_O_3_ (~16% at. Ga)	376	847	470	809	433	865

## Supplementary Materials

The following are available online at https://www.mdpi.com/1424-8220/20/11/3142/s1, Figure S1: Gallium/copper concentrations: (a) distribution map of sample S1 for Cu and Ga elements, (b) distribution map of sample S2 for Cu and Ga elements, (c) distribution map of sample S3 for Cu and Ga elements.
