# Supplementary material for: CuO-Ga2O3 Thin Films as a Gas-Sensitive Material for Acetone Detection
Source: Sensors (Basel). 2020 Jun 2;20(11):3142. doi: 10.3390/s20113142 (PMC7309060; doi:10.3390/s20113142)
Supplement: Supplementary file 1 [file sensors-20-03142-s001.pdf]

Supplementary Materials for CuO-Ga_2O_3 Thin Films as a Gas-sensitive Material for Acetone Detection

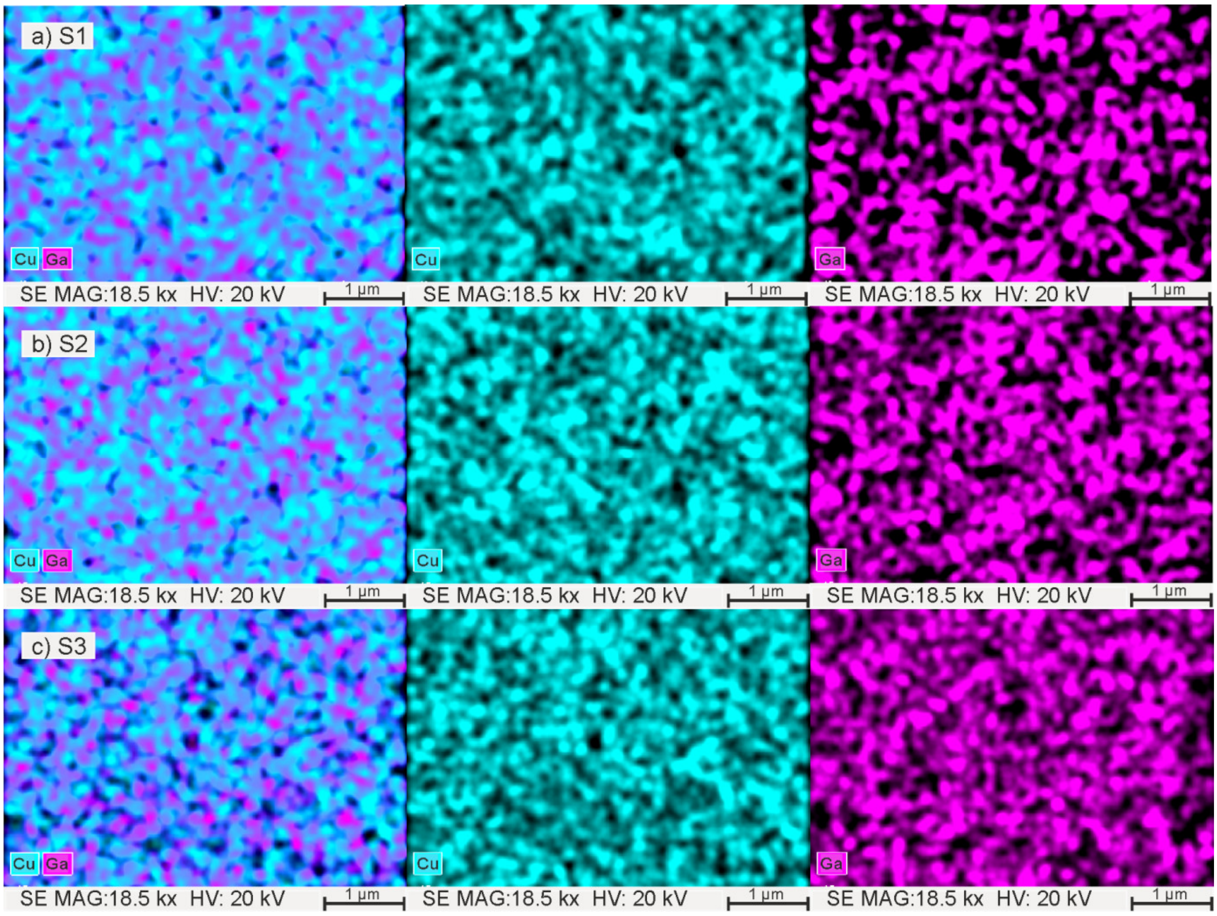

Figure S1. Gallium/copper concentrations: (**a**) distribution map of sample S1 for Cu and Ga elements, (**b**) distribution map of sample S2 for Cu and Ga elements, (**c**) distribution map of sample S3 for Cu and Ga elements.
